# Observational Conditioning in Flower Choice Copying by Bumblebees (*Bombus terrestris*): Influence of Observer Distance and Demonstrator Movement

**DOI:** 10.1371/journal.pone.0088415

**Published:** 2014-02-07

**Authors:** Aurore Avarguès-Weber, Lars Chittka

**Affiliations:** Biological and Experimental Psychology, School of Biological and Chemical Sciences, Queen Mary University London, London, United Kingdom; Universidade de São Paulo, Faculdade de Filosofia Ciências e Letras de Ribeirão Preto, Brazil

## Abstract

**Background:**

Bumblebees use information provided inadvertently by conspecifics when deciding between different flower foraging options. Such social learning might be explained by relatively simple associative learning mechanism: the bee may learn to associate conspecifics with nectar or pollen reward through previous experience of foraging jointly. However, in some studies, observers were guided by choices of ‘demonstrators’ viewed through a screen, so no reward was given to the observers at the time of seeing other bees’ flowers choice and no demonstrator bee was present at the moment of decision. This behaviour, referred to observational conditioning, implies an additional associative step as the positive value of conspecific is transferred to the associated flower. Here we explore the role of demonstrator movement, and the distance between observers and demonstrators that is required for observation conditioning to take place.

**Methodology/Principal Findings:**

We identify the conditions under which observational conditioning occurs in the widespread European species *Bombus terrestris*. The presence of artificial demonstrator bees leads to a significant change in individual colour preference toward the indicated colour if demonstrators were moving and observation distance was limited (15 cm), suggesting that observational conditioning could only influence relatively short-range foraging decisions. In addition, the movement of demonstrators is a crucial factor for observational conditioning, either due to the more life-like appearance of moving artificial bees or an enhanced detectability of moving demonstrators, and an increased efficiency at directing attention to the indicated flower colour.

**Conclusion:**

Bumblebees possess the capacity to learn the quality of a flower by distal observation of other foragers’ choices. This confirms that social learning in bees involves more advanced processes than simple associative learning, and indicates that observational conditioning might be widespread in pollinating insects, raising intriguing questions for the underlying mechanisms as well as the spread of social information in pollinator-plant interactions.

## Introduction

In a variable and complex environment, animals have to constantly update information about resources, threats or mating opportunities. Information can be acquired through potentially costly individual trial-and-error sampling. Alternatively, information can be gathered from the observation of other individuals inadvertently providing valuable information [1]–[3]. Social learning is widespread in animals from primates to insects [4]–[6].

Social insects are particularly appealing as study cases for social learning phenomena. Their complex societies require information transfer among workers to achieve a fluid self-organisation despite the lack of central decision makers. The spread of socially acquired information is favoured by a number of social interactions within the often populous colonies. Social insects can actively advertise a valuable food source to nestmates through scent marks or, in the case of honeybees, through a sophisticated dance ‘language’ [7], [8]. The capacity to provide, within the nest, information about the precise location of a food source seems restricted to honeybees and stingless bees among pollinator species [9], [10].

Bumblebees do not have the dance language to communicate actively about valuable food sources location although they can alert conspecifics about food availability [9], [11]. However, bumblebee foragers do use information inadvertently provided by conspecifics to choose between flowers [5]. Both laboratory [12]–[17] and field experiments [18] demonstrate that bumblebees are attracted by conspecifics and tend to land preferentially on occupied flowers when the flower type is as yet unfamiliar and the conspecifics density is low.

In these cases, the underlying mechanisms could be relatively simple. An innate attraction to conspecifics would be sufficient to lead a bee to land and therefore sample the associated flower. Alternatively, the attractive value of conspecifics could be acquired through a simple associative Pavlovian mechanism between a conspecific and a reward in (accidental) co-feeding occurrences on the same flower patches. The importance of associative learning in social learning behaviour is suggested by the capacity of bumblebees to modulate the response to social cues through experience: the preference for flowers occupied by conspecifics might be promoted when conspecific presence is a reliable predictor of the reward [19]. In a similar vein, the repellent effect of bee scent marks indicating previously visited, and therefore typically unrewarding, flowers [20] seems to be the consequence of past associative experience [21], [22].

However, in some experiments performed on the North American bumblebee species *Bombus impatiens*, and the European *Bombus terrestris* [23], [24], social learning skills cannot be explained by simple associative learning. Study subjects were allowed to observe conspecifics visiting one colour of flowers, but not an alternative colour, and subjects were separated from demonstrators by a screen [23], [24]. Subjects were found to follow conspecifics’ choice and preferred to land on the demonstrator-indicated flower colour over the other flower type in a subsequent testing phase. The flower choice in the test situation follows the act of observing conspecific foraging with a delay. Decisions are thus made without the previous possibility for direct sampling of the flowers or direct interaction with demonstrators; therefore simple attraction to conspecifics could not account for the result. This form of social learning is often described as observational conditioning [25], a higher-level form of associative learning. In this case, an additional associative step is required. The acquired positive value of conspecifics through co-feeding occurrences should be transferred to the associated stimulus in the observation phase. A mechanism based on a two-step association (second-order conditioning [26]) is a likely explanation of bumble bees performance [23].

In this study, we further explore this phenomenon in the European species *Bombus terrestris*. We investigate the factors that determine observers’ attention toward demonstrators and the flower colours on which they can be seen, namely the distance of artificial demonstrator bees from the observing bees, and the movement of demonstrators.

## Results

All tested bees from two colonies preferred to visit blue artificial flowers over yellow flowers prior to any contact with these colours, consistent with innate attraction of bumblebees to blue flowers observed in previous studies (e.g. [27], [28]). The proportion of choices for the blue discs was 65.8±1.7% (mean ± SEM) which was significantly above chance (Chi-square test: n = 51; χ^2^_1_ = 18.3, p<0.001). The colony origin of the tested individuals did not significantly influence the result (independent samples t-test: t_49_ = 0.22, p = 0.83).

**Figure 1:**
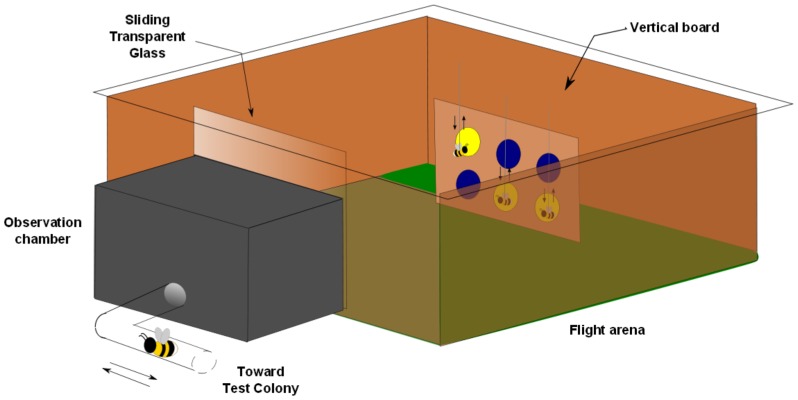
Experimental setup. The observation chamber was connected to the main flight arena through a sliding transparent Plexiglas sheet (only source of illumination). The chamber faced a vertical cardboard on which six coloured discs (blue and yellow) were displayed. The test bumblebees were first individually tested for their naive preference by recording the number of choices for each coloured disc for five minutes. Each test bee was subsequently held in the observation chamber on its outbound journey towards the flight arena, while artificial bees were presented in front of the yellow disks during the 10 minute observation phase. Demonstrator bees were then removed from the arena and the spatial arrangement of the coloured disks on the presentation board was modified. The test bee was finally released into the arena and the number of cumulative choice for each colour was recorded for five minutes. The diagram is not true to scale.

**Figure 2:**
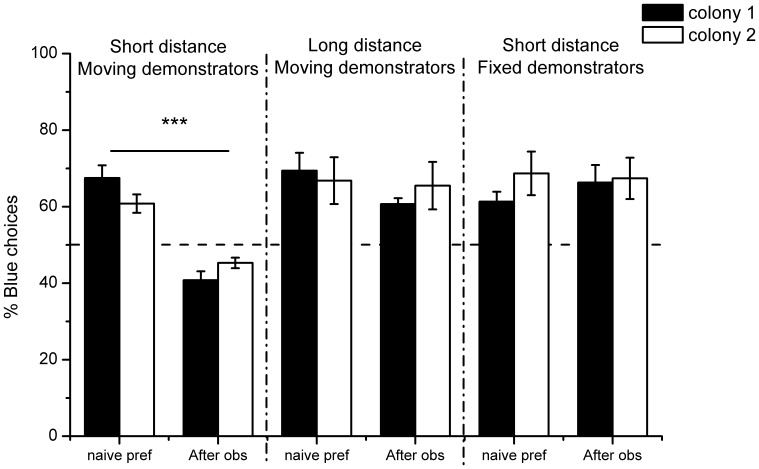
Experimental results. Percentage of choices (mean ± SEM) for the blue colour vs. yellow colour in the non-rewarding tests. The dashed line indicates random choice level. White and black bars show results from individuals of respectively the first and second colony used in this experiment. There was no significantly influence of the colony origin of the tested individuals. Within each treatment, the bars on the left correspond to the naive preference of the test bees without prior exposure to these colours. The bars on the right present colour preference of the same bees after the observation period in which they observed artificial bees displayed in front of the yellow stimuli. The observation period only had a significant influence on bees’ colour preference if the artificial bees were moving and presented as short distance (15 cm) from the observation chamber during the observation phase.

The same bees were tested again for colour preference in a within-individual experimental design after having been given the opportunity to observe model conspecifics in the main flight arena by a transparent Plexiglas sheet (Fig.1). During the observation phase, artificial bees were displayed on the non-preferred yellow artificial flowers while the blue flowers stayed clear. Test individuals were separated into three groups that experienced different observation conditions to investigate the influence of demonstrators’ movement and observation distance. Again, the colony origin did not significantly influence performance (repeated measures ANOVA: n = 51: F_1,45_ = 0.28, p = 0.60). A significant influence of the observation phase on bees’ colour preference was observed (F_1,45_ = 19.69, p<0.001), but only in the case of bees seeing moving demonstrators at a short distance (15 cm) (group effect: F_2,45_ = 14.54, p<0.001; Tukey HSD post-hoc tests revealed that only results from this group showed a significant difference before and after the observation phase: *moving demonstrators – short distance*: p<0.001; *moving demonstrators – long distance*: p = 0.55; *fixed demonstrators – short distance (15* *cm)*: p = 0.99). The bees from this group chose blue artificial flowers at 42.8±1.4% after the observation phase in contrast to 64.5±2.2% beforehand (paired-sample t-test: n = 18; t_17_ = 8.66, p<0.001; Fig. 2). Conversely, neither the presence of moving demonstrators at a 30 cm distance (62.8±2.8% of blue choice after observation vs. 68.2±3.6% before; n = 16; t_15_ = 1.76, p = 0.10; Fig. 2) nor the observation of still demonstrators at short distance (66.8±3.4% of blue choice after observation vs. 64.8±3.0% before; n = 17; t_16_ = 0.52, p = 0.61; Fig. 2) significantly influenced subsequent colour preference.

## Discussion

Our current study confirms that bumblebee (*Bombus terrestris*) flower choices can be influenced by the observation of conspecifics’ choice when making a decision between alternative foraging options [23], as found also previously in another insect species, the North American *B. impatiens* [24]. It is thus possible that multiple species of bumblebees, and perhaps other pollinators, can learn by observing conspecific’s behaviour and without direct interaction with a reward or with conspecifics which flower type is likely to provide nectar.

Social learning capacities are well established in bumblebees making foraging choice in presence of conspecifics [5] but the ability to use social information from remote observation requires a higher processing level than simple attraction to conspecifics. In our study, as in the one by Worden & Papaj [24] on a different bumblebee species, the positive value of conspecifics has to be transferred to the visited flowers during the observation phase to account for a subsequent attractiveness of these flowers in the test phase. This ability is known as observational conditioning [25], [29]. A classical example of observational conditioning is the case of laboratory-reared Rhesus monkeys that acquired a fear for snakes after having observed wild-born monkeys acting fearfully in presence of snakes [30], [31]. The tested monkeys did not show any interest in snakes before the experiment but were particularly agitated and displayed fear when presented with snake after this observation phase. But what remains unclear in this example as well as in the case of bumblebees is the question of whether the response by observers is to some extent guided by an innate preparedness to attach special salience to certain conspecific behaviour patterns [25], [32].

For a better understanding of the relevance of observational conditioning in nature, it is important to explore the conditions under which it occurs. One possibility is that an observation of the foraging behaviour of conspecifics involving flight movements, landing and flower handling might be necessary. Alternatively, only conspecifics’ visual pattern presented on a flower (or a subset of it, e.g. specific colours or striped patterns) may be sufficient, when visually associated with a flower, to promote foraging behaviour of the observing bees toward similar flowers. Our finding that only moving model bees mimicking hovering behaviour promote observational conditioning in bumblebees argues in favour of the first option. However, moving objects in the bees’ visual field have enhanced detectability irrespective of whether the moving items are other pollinators, and might simply attract attention towards the location of movement. Bees are indeed particularly sensitive to movement [33], [34] and bee’s object detection is improved by target movement in the foraging context [35]. Further studies should thus determine the importance of the demonstrator bee’s visual appearance in triggering social learning behaviour.

The question of detectability of demonstrators on flowers is crucial to evaluate the potential ecological impact of observational social learning. Our study shows that conspecific foraging behaviour can have a significant influence within a short- range distance (less than 30 cm) and therefore would likely occur only within flower patches and could not thus attract individuals to a distant foraging patch.

Finally, the confirmation that artificial bee models are efficient demonstrators so long as they exhibit movement has practical implications [13], [24]. Experiments based on live demonstrators are more difficult to control as they involve a training phase for the demonstrators and require the concomitance of foraging motivation in both demonstrators and observers. The information supplied to the test bees might be less reliable due to the variability of demonstrators’ behaviours. In addition, the manipulation of the validity of social information for experimental purposes and a better investigation on the underlying mechanisms could be rendered possible by the use of artificial model bees that can easily be associated with a low reward, empty flowers, or even aversive substances [23]. The experimental use of artificial bees thus opens perspectives toward deeper investigations of the characteristics of social learning behaviour in bumblebees.

## Methods

Bumblebee (*Bombus terrestris*) colonies were provided by Koppert Biological Systems (Berkel en Rodenrijs, Netherlands). Bees (n = 51) from two different colonies (contributing n = 28 and n = 23 tested individuals) were randomly allocated to one of the three observation conditions. The colonies were housed in wooden nest boxes (28×16×11 cm) connected to a flight arena (110×70×30 cm) covered by a UV-transparent Plexiglas ceiling. Light conditions mimicked the natural daylight spectrum and the flicker frequency of strip lights (Activa Daylight Tubes, Osram, Germany) was adjusted by 4.3 kHz ballasts (Philips, Netherlands) to levels beyond bumblebee’s flicker fusion frequency [33], [36]. The nest boxes and the flight arenas were connected via a Plexiglas tube with sliding doors, allowing a controlled individual access to the arena. Individual bees were identified by numbered tags or paint marks, and were removed from the colony after testing. Bees were fed daily with pollen provided directly into the nest.

### Setup

The test colony was connected to an observation chamber (20×15×10 cm), itself connected to the main flight arena. The connection between the observation chamber and the arena could be blocked by a transparent Plexiglas sheet (2 mm thick) (Fig. 1). The observation chamber was dimmer than the flight arena, since it received light only indirectly from the main arena through the transparent Plexiglas sheet. This was meant to ensure that observers in the chamber would attend to events in the more brightly lit flight arena. Observers in the chamber could view a brown vertical cardboard (20×15 cm) placed at various distances from the chamber’s transparent window. This vertical board displayed the colour stimuli during observation and testing phase. Prior to the experiment, the test bees were trained as a group to feed from six (two rows of three) small transparent Plexiglas platforms (1.5×1.5×1 cm with a small cavity (Ø 0.5 cm, 0.2 cm in depth) on top to hold fluids, e.g. droplets of sucrose solution) glued onto the board (Fig. 1). Only bees that were seen feeding jointly with other bees for at least three successive foraging bouts and consequently had the opportunity to form an association between conspecifics and a food reward were selected for testing [19], [21].

### Naive Colour Preference Test

Colour naive bumblebees were tested individually. Blue and yellow coloured disks were displayed (Ø = 57 mm) on the vertical board on top of each transparent feeding platform (three disks of each colour were randomly allocated to platforms). Disks were cut from laminated coloured papers. Both colours were easily discriminable from each other and from the brown background (see [23] for details). Feeding platforms offered only water solution. Choices (contact with landing platforms by antennae or feet, or actual landings) were recorded for five minutes. After the test, the bees were allowed to collect sucrose on clear platforms (no associated colour) and return to the hive.

### Observation Phase

At the next emergence from the hive, the same bees were individually held for ten minutes in the observation chamber separated from the arena by a transparent Plexiglas sheet (Fig. 1). The board displayed three yellow and three blue coloured disks randomly allocated to each Plexiglas platform. Three artificial demonstrator bees were positioned in front of each yellow disk. Model bees were shaped using oven-hardening modelling clay (Fimo Soft, Staedtler, Germany) and painted using the following paints: yellow (Rheotech, Canada, Acrylics Bright Yellow) and black (Winsor & Newton, USA, Griffin fast drying oil painting, ivory black). For the white tip of the abdomen, the white modelling clay was left unpainted. Colours were chosen to reflect natural *Bombus terrestris* colour patterns as seen by bumblebees [37], [38].

The tested bees were allocated to three different treatments:

#### Moving demonstrators – long distance

The board was presented at 30 cm from the observation screen. At this distance, demonstrator artificial bees (length 20 mm) subtended a visual angle of 3.8° in the observer’s visual field and should therefore have been near the limit of detection range for targets that move relative to their backdrop [39]. Stationary targets should be difficult or impossible to detect at this angle [39]. Artificial demonstrator bees were moving up and down in front of the yellow coloured discs to mimic a bee in approach flight to the flower, or hovering in front of it. The model bees were attached to a transparent string (fishing line) and moved vertically in sinusoidal movements (amplitude 33 mm; 33 cycles per minute) by a custom-built motorised device (Fischertechnik GMBH, Waldachtal, Germany).

#### Moving demonstrators – short distance

The board was presented at 15 cm from the observation screen (visual angle: 7.6°). Demonstrator bees were moving in front of the yellow disks as described above.

#### Fixed demonstrators – short distance

The board was presented at 15 cm from the observation screen. Demonstrator bees were fixed in front of the yellow disks and remained stationary during the entire observation phase.

### Preference Test following the Observation Phase

After the ten minutes observation period, the artificial demonstrator bees were removed from the flight arena and a novel spatial arrangement of the six coloured disks was presented to the test bees at the same distance. The disks and feeding platforms were cleaned with ethanol before the testing phase. The tests were non-rewarding; feeding platforms contained only water. The test bee was then released into the main chamber by sliding open the Plexiglas window connecting the observation chamber to the arena (Fig. 1). The bee’s choices for each coloured stimulus were recorded during five minutes.
